# Subglacial swamps

**DOI:** 10.1098/rspa.2014.0340

**Published:** 2014-11-08

**Authors:** T. M. Kyrke-Smith, A. C. Fowler

**Affiliations:** 1 Department of Earth Sciences, University of Oxford, Oxford, UK; 2 OCIAM, University of Oxford, Oxford, UK; 3 MACSI, University of Limerick, Limerick, Republic of Ireland

**Keywords:** subglacial hydrology, canals, Smith-Bretherton instability, sediment-floored channels

## Abstract

The existence of both water and sediment at the bed of ice streams is well documented, but there is a lack of fundamental understanding about the mechanisms of ice, water and sediment interaction. We pose a model to describe subglacial water flow below ice sheets, in the presence of a deformable sediment layer. Water flows in a rough-bedded film; the ice is supported by larger clasts, but there is a millimetric water layer submerging the smaller particles. Partial differential equations describing the water film are derived from a description of the dynamics of ice, water and mobile sediment. We assume that sediment transport is possible, either as fluvial bedload, but more significantly by ice-driven shearing and by internal squeezing. This provides an instability mechanism for rivulet formation; in the model, downstream sediment transport is compensated by lateral squeezing of till towards the incipient streams. We show that the model predicts the formation of shallow, swamp-like streams, with a typical depth of the order of centimetres. The swamps are stable features, typically with a width of the order of tens to hundreds of metres.

## 1. Introduction

Glaciers and ice sheets commonly have basal temperatures that reach melting point, on account of the geothermal heat flux from the Earth, and the insulating effect of the ice cover; consequently, most glaciers and ice sheets have a basal water system that is formed from the resulting meltwater. In glaciers, this can be enhanced by percolation of surface meltwater to the bed, whereas in the interior of ice sheets the basal meltwater is formed entirely by subglacial melting.

It is widely acknowledged that the resulting subglacial hydrologic system exerts a fundamental control on ice sheet dynamics; the volume of evidence for an active subglacial water system in Antarctica and under ice masses is constantly growing (e.g. [1,2]). The large variation in ice velocities across Antarctica, and particularly the presence of ice streams, is commonly considered to be a consequence of this subglacial hydrologic system (e.g. [3,4]). Furthermore, it is important to know about water movement at the bed because of its critical role in phenomena such as potentially hazardous subglacial flooding (e.g. [5]), as well as in glacial erosion and landform evolution (e.g. [6,7]). An improved understanding of the drainage system will allow us to learn more about critical feedbacks between the water, ice and bed.

Subglacial drainage systems commonly discussed in the literature, such as linked cavities [8,9], thin patchy films [10] and Röthlisberger channels [11] are framed in the context of the classic hard glacial bed: cavities/thin patchy films form behind bedrock protrusions, while the channels are mostly thought of as rock-floored and incised into the ice, and the issue of sediment transport through them is rarely discussed (cf. Fowler [12, p. 699 ff].). However, evidence suggests that the observed rapid basal velocities in parts of Antarctica are facilitated by the presence of a layer of till at the base of the sheet [13–16], and therefore these hard-bed drainage systems may not be relevant to subglacial water flow beneath ice streams. A soft bed has rather become the paradigm assumption.

For probable permeabilities and till thickness, Darcian porous flow through the sediment is likely to be insufficient to evacuate all the meltwater present at the bed [17,18], and as such the till is water saturated. As a granular material, the till is expected to deform with Coulomb-plastic behaviour [16,19–22], and its behaviour depends on the effective pressure, which is determined by the drainage system. In an effort to understand the possible nature of subglacial water flow over a layer of saturated till, Walder & Fowler [18] developed the concept of canals, which are in essence inverted Röthlisberger channels, incised in the underlying sediment. In their work, they showed that these canals would form a distributed system owing to the fact that effective pressure decreased with increasing water flux. However, their model can be criticized on a number of counts. For example, they assumed a pseudo-viscous flow law for till, an assumption that has led to debate [23–25], and on which an effective conclusion has yet to be reached. More pertinently, Walder and Fowler's model was conceptually similar to that of Röthlisberger, insofar as it assumed a local channel surrounded by till and ice, and the resulting model was consequently essentially algebraic in nature. It also made assumptions concerning the shape of channels. One was that the canal depth was controlled by the necessity to enable bedload transport to remove sediment. The other was that whether a canal or channel formed depended on the relative softness of ice and till, and this depended on the effective pressure.

The aim of this paper is to develop a more sophisticated model of subglacial water flow interacting with the sediment in the presence of a soft bed. In particular, we abandon the restrictive view of channels or canals as pre-formed, and we aim to describe their evolution from an initial spatially extensive rough water film. As an eventual aim, we hope to characterize sub-ice-stream water flow sufficiently to allow for a useful parametrization of its effect on the ice flow. This requires a suitable averaging over the presumably small-scale dimensions of hydraulic stream flow to describe the larger scale ice stream flow. This would be a significant improvement on recent work looking at ice-stream formation over a Coulomb-plastic till, which simply uses a basic diffusion equation to describe the evolution of a water layer over saturated till, assuming the water diffuses from high-to-low effective thickness [26–28]. Furthermore, other water flow models that currently exist on the larger scale generally consist of a simple distributed system for the water, where the water flows down gradients in hydraulic potential (e.g. [29,30]). While such models are able to demonstrate where we expect the water to flow, and the sensitivities of the flow paths to small changes in the system, they do not begin to include any of the intricacies associated with the subglacial hydrologic system. There have been various efforts made at forming more detailed hydrology models in the literature, some considering simple flow-band models [31,32], and others with two-dimensional models including the details of interaction between a distributed drainage system and a channellized drainage system (e.g. [33–35]). For these latter models, the channellized drainage is on the grid scale. In this paper, however, we look at modelling on the small scale, with a view to upscale later to model the water flow on the ice-sheet scale.

In §2, we present the coupled model for water and sediment transport that forms the basis of our study. We non-dimensionalize the system in §3, before simplifying and solving it in §3a. A discussion of the results and their context follows in §4, and the conclusions of the study are in §5.

## 2. Set-up and governing equations

We conceive of water at the base of an ice stream in the following way. Water is formed by the melting of ice at grain boundaries, whence it seeps down to the bed in a steady drizzle of some 5 mm yr^−1^. The ice is underlain by sediments, or till, which thus become saturated with water. Typically, the sediments are relatively impermeable, and this has two effects: the water cannot drain through the sediments and must consequently form some kind of hydraulic system between the ice and the bed. The second consequence is that this causes the water to be at high pressure, so that the till is deformable. The simplest kind of water system is a uniformly distributed thin film at the interface between the ice and the till. With a clean separation, as imagined by Weertman [36], the resulting flow is always unstable [37], but we can suppose such a film could exist if it is thinner than supporting clast sizes; we call this a *Creyts–Schoof* film [38]. The idea is that a thin water film whose thickness is measured in millimetres will generally not drown the coarser particles of the till, so that despite the partially lubricating effect of the water, the ice still grips the coarse till and can transmit a stress to it. Evidently, the degree to which this happens depends on the thickness of the water film. The film is subject to Walder's [37] instability mechanism, and we can expect some kind of streams to form. Our model will show this.

**Figure 1.**
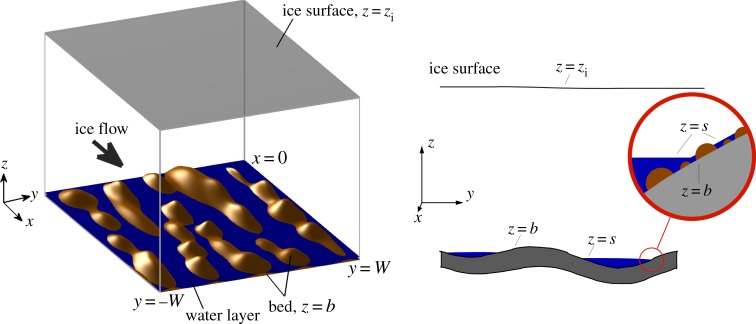
Geometry of the flow under consideration. A three-dimensional schematic and a cross section with notation labelled.

We consider a cartesian set of axes (*x*,*y*,*z*) in which *x* points in the ice flow direction, *y* is across flow and *z* is vertically upwards. As shown in figure 1, the till surface is denoted by *z*=*b*, the lower ice surface is denoted by *z*=*s* and the upper ice surface is denoted by *z*=*z*_i_, all of these being functions of *x*, *y* and time *t*. The depth of the water film is thus

2.1 h=s−b.

The hydraulic head driving water flow at the ice/till interface is

2.2 $$\psi =p_{w}+\rho_{w}gs-(p_{a}+\rho_{i}gd_{i}),$$

where *p*_w_ is water pressure, *p*_a_ is atmospheric pressure (assumed constant), *ρ*_w_ and *ρ*_i_ are the densities of water and ice, *g* is the acceleration due to gravity, and *d*_i_ is the local ice depth, a constant and distinct from *z*_i_ which varies in the downstream ice flow direction; the constant term in parenthesis is added for convenience. The ice pressure *p*_i_ is approximately cryostatic, and it is common in analysing ice flow dynamics (as also in numerous other fluid flows) to define a reduced pressure *Π* which is the ice pressure minus its cryostatic component. At the base, this takes the form [6], eqn (2.1),

2.3 $$p_{i}=p_{a}+\rho_{i}g(z_{i}-s)+\Pi ,$$

and thus (cf. [6], eqn (2.8))

2.4 $$\psi =\rho_{i}g(z_{i}-d_{i})+\Delta \rho_{\mathrm{wi}}gs-N+\Pi -\tau_{\mathrm{nn}},$$

where *τ*_nn_ is the deviatoric normal stress of the ice,

2.5 $$\Delta \rho_{\mathrm{wi}}=\rho_{w}-\rho_{i}$$

and

2.6 $$N=-\sigma_{\mathrm{nn}}-p_{w}=p_{i}-p_{w}-\tau_{\mathrm{nn}}$$

is the effective normal stress at the ice/sediment interface. Equation (2.4) reflects the well-known fact that basal water flow is driven primarily by the ice surface slope, and the basal slope only contributes 9% to the flow direction (because Δ*ρ*_wi_≈0.09*ρ*_i_). In the following subsections, we describe briefly the constituent parts of the model involving ice, water and sediment motion, together with the important concept of a closure relation.

### (a) Ice flow

Fowler [39,6] derived expressions for the solution of the problem of ice flow over locally varying small amplitude topography. If *x* denotes the direction of flow, then we split the ice surface elevation into the sum of a local average value and the perturbation from this; specifically, we write

2.7 $$z_{i}=d_{i}Z+d_{i}S_{i}H,$$

where

2.8 $$S_{i}=-d_{i}Z^{\prime }$$

is the (small) average ice upper surface slope, *Z*∼*O*(1) is the dimensionless local average depth and *H* is a dimensionless measure of the local ice surface perturbation due to flow over any short wavelength basal topography. Typically, we suppose *Z* varies on length scales of hundreds of kilometres, while *H* varies on scales of hundreds of metres. It follows that the hydraulic potential is

2.9 $$\psi =\rho_{i}gd_{i}(Z-1)+\Delta \rho_{\mathrm{wi}}gs-N+\Phi ,$$

where *Φ* is defined as

2.10 $$\Phi =\Pi -\tau_{\mathrm{nn}}+\tau_{b}H,$$

and the (average) basal stress is

2.11 $$\tau_{b}=\rho_{i}gd_{i}S_{i}$$

(note that *Φ*=−*Θ* in the notation of Fowler [6]). The hydraulic potential is defined up to the addition of an arbitrary function of time, and we will use this later.

We are able to solve the ice flow problem over relatively small length scales, typical of those which will concern us here. The method is as follows: the ice flow equations are those of slow flow, and if we assume a constant viscosity, they reduce to the equations of Stokes flow, which are linear. In addition, the ice upper and lower surfaces are small perturbations of level surfaces, so that the boundary conditions on them can be linearized about the unperturbed flat surfaces. The consequence of this is that we obtain a linear problem with inhomogeneous boundary conditions on upper and lower surfaces, which can therefore be explicitly solved by taking a Fourier transform in the *x*-direction, and solving the resulting linear differential equations analytically in the vertical.

The boundary conditions are those of no stress at the upper surface, while at the base the sliding law provides a relation between basal downstream velocity, shear stress and effective stress *N*. If we suppose that the normal stress at the base, and more specifically *Φ*, in (2.10), is given then the Fourier transforms of all other quantities at the upper and lower surfaces can be written ultimately in terms of the Fourier transforms $\hat{\Phi },\hat{N}$ of *Φ* and *N*.

Specifically, the upper and lower surface kinematic conditions take the form

2.12 $$\begin{array}{rl} H_{t} & =v(H,\Phi ,N)\\ \text{and}\;s_{t}+us_{x} & =w(H,\Phi ,N), \end{array}\}$$

where in both conditions, we can ignore small source terms due to surface accumulation and basal melting, respectively. Here, *u* is the ice velocity in the *x*-direction (essentially constant), and *v*,*w* are the vertical velocities at surface and base, respectively.

It turns out that the ice surface perturbation *H* relaxes rapidly to a quasi-equilibrium, so that *H* can be determined, and *w* depends only on *Φ* and *N* and primarily the former. Specifically, we have, approximately,

2.13 $$w=\frac{1}{\eta_{i}}\int_{R^{2}}J(x-\xi )\Phi (\xi )\;d\xi ,$$

where

2.14 $$\hat{J}=\frac{1}{M(j)k},\;j=kd_{i},$$

where $\hat{J}$ is the Fourier transform,

2.15 $$\hat{J}(k)=\int_{R^{2}}J(x)\;e^{ik\cdot x}\;dx,$$

and *k*=|**k**|. Note that the quantities, particularly **x** and **k**, are dimensional in these definitions (which have been translated from the dimensionless equivalents in [6]), but *M* is dimensionless and is shown in fig. 3 of [6]. Particularly, for wavelengths less than about three times the ice depth ($2\pi /k≲\pi d_{i}$, thus j≳2), *M*≈2 is constant. Note that (2.13) ignores a similar integral term due to sliding and thus *N*, which is in fact negligible for j≳2. If we presume the short wavelength limit in which *M*≈2, then a direct calculation shows that

2.16 $$J(x)=\frac{1}{\pi |x|}.$$

A rather better approximation which takes account of the fact that $\hat{J}$ is finite (and specifically $∼\frac{1}{4}d_{i}$) as *k*→0 is to choose

2.17 $$J(x)=\frac{e^{-k^{*}|x|}}{\pi |x|},\;k^{*}=\frac{4}{d_{i}},$$

since then

2.18 $$\hat{J}=\frac{1}{{\left(k^{2}+k^{*2}\right)}^{1/2}}.$$

This means that the kernel *J*(**x**) in (2.13) decays exponentially rather than algebraically at large wavenumber, so that *w* is insensitive to short wavelength variations of *Φ* (i.e. they are damped).

### (b) Water flow

The base of the ice rests on a cobbled bed of subglacial till, typically having a range of particle sizes varying from coarse clasts to fine sediments. Between the coarser cobbles, meltwater from the ice, produced by the combined effects of viscous heating in the basal ice and geothermal heat flux from below, flows in a thin film which can drown the finer particles, but not the coarsest clasts (unless it becomes quite deep); this is the *Creyts–Schoof* water film [38].

Mass conservation of the water flow in the film takes the form

2.19 $$\frac{\partial h}{\partial t}+\nabla .\;q=\Gamma ,$$

where, on a regional scale,

2.20 $$\Gamma =\frac{G+\tau_{b}u-q_{T}}{\rho_{w}L};$$

*G* is the geothermal heat flux, *τ*_b_ is the basal ice stress, *u* is the basal ice velocity, *q*_T_ is the sensible heat flux into the overlying ice and *L* is the latent heat. In our model, we take *Γ* to be constant because we are concerned with small length scales; *Γ* will of course depend on ice depth and velocity, with its variation occurring over larger length scales. It is important to realize that the frictional heat source *τ*_b_*u* arises from an integration of the ice viscous dissipation term over the basal sliding region, and *τ*_b_ and *u* here refer to conditions near the base of the far-field ice sheet flow, but far from the actual interface itself [40]; they are the quantities used in the ice sheet sliding law.

We assume a local Poiseuille flow in the water film, which implies

2.21 $$q=-\frac{h^{3}}{12\eta_{w}}\nabla \psi ,$$

where *η*_w_ is the viscosity of water; thus *h* satisfies

2.22 $$\frac{\partial h}{\partial t}=\nabla .\;\left[\frac{h^{3}}{12\eta_{w}}\nabla \psi \right]+\Gamma .$$

### (c) Film closure

Equation (2.22) must be supplemented with a further closure equation, which is analogous to the closure equation in Röthlisberger's [11] channel flow model. The reason for this is that the effective normal stress in (2.6) varies rapidly along the Creyts–Schoof film. Clearly, the actual normal stress is equal to the water pressure on those parts of the film which are unsupported, with the excess stress being provided by the supporting clasts. As a consequence, a Röthlisberger-type closure condition acts on the film due to the differential stress along the ice surface. A suitable prescription for this closure equation is of the form

2.23 $$h_{t}=\frac{\rho_{w}\Gamma }{\rho_{i}}-\frac{l^{*}(h)N}{\eta_{i}},$$

where l* is a measure of the distance between supporting clasts. It will depend on film thickness *h* because as the film thickens, the distance between the supporting clasts increases, so that l* is an increasing function of *h*. When the water film becomes deep enough for separation of the bed and ice to occur, the water flows in streams or cavities, and it seems that l* should then become equal to the stream width, or more generally be a non-local functional of *h*. Here we simply define l* as

2.24 $$l^{*}(h)=\frac{l_{0}}{N^{*}(h)},$$

where *l*_0_ is a length scale that represents the typical clast spacing in the absence of water, and N*(*h*) (denoted *Λ*(*h*) in [41]) is some decreasing function of *h*, with N*(0)=1.

### (d) Sediment transport

Sediment transport is most usually discussed in the context of fluvial or aeolian transport, where it is due to the action of the water or wind stress on the sand grains [12]. The resulting evolution of the sediment surface is then described by the *Exner equation*, which expresses conservation of sediment by relating sediment surface elevation to the particle flux. The same situation can occur subglacially, but now the sediment can be transported both by shear in the water film and by shear due to the overlying ice; in addition, there is a squeezing effect caused by horizontal differences in the ice normal stress (and thus also the effective stress).

The evolution of the sediment surface *z*=*b* in our model is thus given by the Exner equation

2.25 $$(1-\phi )b_{t}+\nabla .\;q_{c}+\nabla .\;q_{b}=-E,$$

where *ϕ* is the bed porosity, and the volumetric sediment flux has two constituents, a bedload volume flux **q**_b_ and a creep flux **q**_c_; *E* represents net erosion of the bed as suspended sediment and can also be written as a divergence (of minus the suspended sediment flux). We suppose

2.26 $$q_{c}=(1-\phi )\left\{\frac{1}{2}uAi-\frac{A^{3}}{12\eta_{s}}\nabla N\right\};$$

here **i** is the unit vector in the ice flow direction *x* and *A* is the depth of the deformable till layer. The first term represents the shear-driven deformation by the ice, and the second represents till squeezing by effective pressure gradient. The depth *A* is determined by the horizon below which the effective pressure (which increases with depth) is sufficiently large that the yield stress is not exceeded. It is given by Fowler [6] as

2.27 $$A=\frac{{\left[\tau /\mu -N\right]}_{+}}{\Delta \rho_{\mathrm{sw}}g(1-\phi )},$$

where

2.28 $$\Delta \rho_{\mathrm{sw}}=\rho_{s}-\rho_{w},$$

*ρ*_s_ being the sediment density and *μ* is the coefficient of interparticle friction. Note that *τ* in (2.27) is the actual shear stress at the interface and not the same as the ‘far-field’ basal stress *τ*_b_ used in the sliding law. The two are related [42], if we suppose that the actual sliding law at the interface is

2.29 τ=f(u,N),

by the fact that

2.30 $$\tau_{b}=\bar{f(u,N)},$$

where the overline denotes a local spatial average. In particular, if we choose

2.31 $$\tau =cu^{p}N^{q},$$

then, since *u* is not perturbed at leading order at the interface,

2.32 $$\tau =\frac{\tau_{b}N^{q}}{{\bar{N}}^{q}}.$$

The bedload transport is defined in terms of an effective stress in the water layer, which combines the stress exerted by the water flow,

2.33 $$\tau_{w}=-\frac{1}{2}h\nabla \psi ,$$

with the effect of the bed slope. The effective stress is [6]

2.34 $$\tau_{e}=-\frac{1}{2}h\nabla \psi -\Delta \rho_{\mathrm{sw}}gD_{s}\nabla b,$$

where *D*_s_ is the grain size of the sediment. We then write

2.35 $$q_{b}=(1-\phi )B\tau_{e},$$

where *B* is generally a function of *τ*_e_. Typical forms (e.g. the Meyer-Peter/Müller Law) involve measurements on turbulent flows, together with absence of transport below some critical yield stress, thus *B*∝[*τ*_e_−*τ*_c_]^1/2^_+_, but such sub-aerial relations may be inappropriate, since for thin films and relatively slow flows, we can expect the flow to be laminar and not well described by sub-aerial formulae. Note, however, the specific requirement that *B*=0 when *τ*_w_=0, i.e. *h*=0.

Normally, one assumes the bedload layer to be a very thin layer, one or two grains thick, at the base of a deeper water flow. More precisely, the bedload is a constituent of the fluid flow, so that the water flux is really considered to comprise both water and mobile sediment. In particular, we must have *q*_b_<*q* (typically, it is much smaller).

The model for coupled ice, water and sediment evolution thus consists of the seven equations (2.1), (2.9), (2.12), (2.13), (2.22), (2.23) and (2.25) for *h*, *s*, *b*, *ψ*, *Φ*, *w* and *N*.

### (e) Boundary conditions

To pose suitable boundary conditions, we consider a rectangular domain in which the upstream end *x*=0 might be a divide, or a cold-temperate transition line; in either case, it is reasonable to put

2.36 h=0at x=0.

In this case, there can be no hydraulic sediment transport, and so we must choose *B*=0. If the till is not deformable, then *A*=0 and no further condition is necessary. If it is, it seems reasonable that the till flux is just $\frac{1}{2}Aui$, and then we have

2.37 $$\frac{\partial N}{\partial x}=0\;\mathrm{at}\;x=0.$$

Alternatively, we might prescribe an inlet water mass flow

2.38 $$-\frac{h^{3}}{12\eta_{w}}\frac{\partial \psi }{\partial x}=q_{0}\;\mathrm{at}\;x=0,$$

together with (2.37). A suitable choice of water flux is the flux scale

2.39 $$q_{0}=\Gamma l,$$

where *l* is a suitable macroscopic ice flow length scale.

At the sides, it is suitable to prescribe no transverse flux of water or fluvial sediment, and then

2.40 $$\frac{\partial \psi }{\partial y}=\frac{\partial h}{\partial y}=0\;\mathrm{at}\;y=\pm W,$$

where 2*W* is the width of the domain.

Finally, the front of the flow will typically be a land-based terminus or more likely a grounding line, but in either case we may put

2.41 N=0at x=l.

It is less obvious how to prescribe an outflow condition, but one which avoids the possibility of unnecessary boundary layers is to choose

2.42 $$\frac{\partial h}{\partial x}=0\;\mathrm{at}\;x=l,$$

although other justifiable choices are possible.

## 3. Non-dimensionalization

We non-dimensionalize the equations by scaling the variables as

3.1 $$\begin{array}{rl} & h∼h_{0},\;s∼d,\;b∼h_{0},\;t∼t_{0},\;B∼B_{0},\\ & \tau_{e}∼\tau_{0},\;A∼2d_{T},\;\psi ∼\rho_{i}gd_{i},\;J∼\frac{1}{l},\;S_{i}=\frac{d_{i}S}{l},\\ & x∼l,\;N∼N_{0},\;\Phi ∼N_{0}\;u∼u_{0},\;l^{*}=\frac{l_{0}}{N^{*}(h)}, \end{array}\}$$

where the subsidiary scales are defined by (2.39), and

3.2 $$\begin{array}{rl} B_{0} & =\frac{q_{b}}{\tau_{0}},\;h_{0}={\left(\frac{12\eta_{w}\Gamma l^{2}}{\rho_{i}gd_{i}}\right)}^{1/3},\;\tau_{0}=\frac{\rho_{i}gd_{i}h_{0}}{2l},\;N_{0}=\frac{\rho_{i}gd_{i}^{2}}{l},\\ t_{0} & =\frac{h_{0}l}{u_{0}d_{T}},\;d=\frac{N_{0}}{\Delta \rho_{\mathrm{wi}}g},\;d_{T}=\frac{N_{0}}{\Delta \rho_{\mathrm{sw}}g(1-\phi )}, \end{array}\}$$

the last two being from Fowler [6].

The value of the bedload transport *q*_b_ is estimated as follows. Values of *q*_b_/*q*_0_ for a turbulent Alpine proglacial stream are of order 10^−4^ [43]. A similar assumption under an ice sheet gives the estimate in table 1. As a typical bimodal material, there is no value for the grain size *D*_s_. Tulaczyk *et al*. [44] give values of approx. 100 *μ*m for the sandy till under Whillans Ice StreamQ1. Our value is comparable to this, but somewhat reduced so that the typical film water stress ∼*ρ*_i_*ghS* is slightly greater than the critical Shields stress approx. 0.06Δ*ρ*_sw_*gD*_s_ necessary for transport to occur. Values of the other scales are given in table 2.

**Table 1.** Assumed constants in the model.

symbol	meaning	typical value
*B*_0_	sediment transport coefficient	*q*_b_/*τ*_0_
*d*_i_	ice depth	10^3^ m
*D*_s_	grain size of sediment	30 μm
*g*	gravity	9.8 m s^−2^
*l*	ice sheet length scale	500 km
*l*_0_	clast spacing	1.2 m
*q*_b_	bedload transport	0.3 m^2^ yr^−1^
*S*_i_	ice surface slope	∼10^−3^
*u*_0_	ice velocity	100 m yr^−1^
*Γ*	melt rate	3 mm yr^−1^
Δ*ρ*_sw_	*ρ*_s_−*ρ*_w_	1.6×10^3^ kg m^−3^
Δ*ρ*_wi_	*ρ*_w_−*ρ*_i_	83 kg m^−3^
*η*_i_	ice viscosity	10^14^ Pa s
*η*_w_	water viscosity	1.8×10^−3^ Pa s
*η*_s_	notional sediment viscosity	2.7×10^9^ Pa s
*μ*	coefficient of friction	0.6
*ρ*_i_	ice density	0.9×10^3^ kg m^−3^
*ρ*_w_	water density	10^3^ kg m^−3^
*ρ*_s_	sediment density	2.6×10^3^ kg m^−3^
*ϕ*	sediment porosity	0.4

**Table 2.** Variable definitions and associated scales if appropriate.

symbol	meaning	scale
*A*	till depth	
*b*	sediment elevation	
*d*	sediment topography	21.6 m
*d*_T_	till deformation depth	1.87 m
*h*,*h*_0_	water film thickness	3.9 mm
*l*_D_	drumlin length scale	625 m
l*,*l*_0_	distance between supporting clasts	1.2 m
*N*,*N*_0_	effective stress scale	1.76×10^4^ Pa
*q*,*q*_0_	water flux	1.5×10^3^ m^2^ yr^−1^
*s*	ice/water interface	
*t*,*t*_0_	time	10.4 yr
*z*_i_	upper ice surface	
*Π*	reduced pressure	
*τ*_nn_	deviatoric normal stress at bed	
*τ*_b_	basal shear stress	0.88×10^4^ Pa
*τ*_w_,*τ*_0_	water shear stress	3.4×10^−2^ Pa
*ψ*	hydraulic potential	

This leads to the dimensionless set of equations

3.3a $$\begin{array}{rl} h & =\frac{s}{\delta }-b, \end{array}$$

3.3b $$\begin{array}{rl} \varepsilon h_{t} & =\nabla .\;[h^{3}\nabla \psi ]+1, \end{array}$$

3.3c $$\begin{array}{rl} \psi & =Z+\nu (s-N+\Phi ), \end{array}$$

3.3d $$\begin{array}{rl} \frac{1}{\delta }s_{t}-h_{t}+\nabla .\;[Aui-\beta A^{3}\nabla N] & =\sigma \nabla .\;\left[B\left\{h\nabla \psi +\lambda \nabla \left(\frac{s}{\delta }-h\right)\right\}\right], \end{array}$$

3.3e $$\begin{array}{rl} \varepsilon rh_{t} & =1-\frac{\Pi N}{N^{*}(h)} \end{array}$$

3.3f $$\begin{array}{rl} \text{and}\;\;\Omega s_{t}+us_{x} & =\Lambda \int_{R^{2}}J(x-\xi )\Phi (\xi )\;d\xi , \end{array}$$

where the parameters are defined by

3.4 $$\begin{array}{rl} \varepsilon & =\frac{u_{0}d_{T}}{q_{0}},\;\nu =\frac{d_{i}}{l},\;\delta =\frac{h_{0}}{d},\;\lambda =\frac{2\Delta \rho_{\mathrm{sw}}D_{s}}{\rho_{i}d_{i}},\;\sigma =\frac{q_{b}}{u_{0}d_{T}},\\ r & =\frac{\rho_{i}}{\rho_{w}},\;\Pi =\frac{\rho_{i}l_{0}N_{0}}{\rho_{w}\Gamma \eta_{i}},\;\Omega =\frac{d_{T}}{h_{0}},\;\Lambda ={\left(\frac{l}{l_{D}}\right)}^{2},\;\beta =\frac{2d_{T}^{2}N_{0}}{3\eta_{s}lu_{0}}, \end{array}\}$$

where *l*_D_ is the drumlin length scale [42]

3.5 $$l_{D}={\left(\frac{\eta_{i}u_{0}}{\Delta \rho_{\mathrm{wi}}g}\right)}^{1/2}.$$

The dimensionless deformable till thickness is, if we assume (2.32),

3.6 $$A=\frac{1}{2}{\left[\frac{SN^{b}}{\mu {\bar{N}}^{b}}-N\right]}_{+}.$$

If we take the kernel to have the form of (2.17), then its dimensionless expression is

3.7 $$J=\frac{e^{-a|x|}}{\pi |x|},\;a=\frac{4l}{d_{i}}.$$

Typical values of the parameters are given in table 3, with values of variable scales given in table 2.

**Table 3.** Approximate dimensionless parameter values.

symbol	typical value
*a*	2×10^3^
*r*	0.9
*α*	6.2
*β*	0.96×10^−5^
*γ*	1.33
*δ*	1.8×10^−4^
*ε*	0.125
λ	1.1×10^−7^
*Λ*	0.64×10^6^
*ν*	2×10^−3^
*Π*	2.0
*σ*	1.6×10^−3^
*Ω*	0.48×10^3^

## 4. Simplification and reduction

The basic model in () can be reduced to consider the leading-order effects of interest for studying the water–sediment interaction in more detail. We define

4.1a Ψ=s−N+Φ;

then, bearing in mind that *Z*′(*x*)=−*S*, we have

4.1b $$\begin{array}{rl} \varepsilon h_{t}+{\left(Sh^{3}\right)}_{x} & =\nu \nabla .\;[h^{3}\nabla \Psi ]+1, \end{array}$$

4.1c $$\begin{array}{rl} \frac{1}{\delta }s_{t}-h_{t}+\nabla .\;[Aui-\beta A^{3}\nabla N] & =-\sigma {\left(BSh\right)}_{x}+\sigma \nabla .\;\left[B\left\{\nu h\nabla \Psi +\lambda \nabla \left(\frac{s}{\delta }-h\right)\right\}\right], \end{array}$$

4.1d $$\begin{array}{rl} \varepsilon rh_{t} & =1-\frac{\Pi N}{N^{*}(h)}, \end{array}$$

4.1e $$\begin{array}{rl} \Omega s_{t}+us_{x} & =\Lambda \int_{R^{2}}J(x-\xi )\Phi (\xi )\;d\xi \end{array}$$

4.1f $$\begin{array}{rl} \text{and}\;A & =\frac{1}{2}{\left[\frac{SN^{b}}{\mu {\bar{N}}^{b}}-N\right]}_{+}, \end{array}$$

providing six equations (along with (4.1a)) for *h*, *s*, *N*, *Ψ*, *Φ* and *A*.

We are interested in the possibility of a lateral, stream-forming instability in which downstream sediment transport is compensated by cross-stream sediment drift. A similar analysis was carried out by Fowler [6], using a model which is a slightly simpler version of (4.1), but his focus was on the interaction of water flow with ice deformation (and the formation of mega-scale glacial lineations (MSGLs)) rather than the formation of genuine finite depth streams. Indeed, his instability results were not pursued beyond the infinitesimal régime.

Our interest here is not with moulding of the subglacial topography, and so we will seek simplifications for the evolution equation (4.1e) for *s*. We might, for example, take the right-hand side to be prescribed.

There is a basic *x*-dependent uniform state in which

4.2 $$\Psi =0,\;N=\frac{N^{*}(h)}{\Pi },\;Sh^{3}=x,$$

whence also *A*=*A*(*x*), and the sediment surface *b*=*s*/*δ*−*h* is given by

4.3 $$b_{t}=\sigma \lambda {\left[Bb_{x}\right]}_{x}-{\left(Au\right)}_{x}+\beta {\left(A^{3}N^{\prime }h_{x}\right)}_{x}-\sigma {\left(BSh\right)}_{x},$$

where *N*′=d*N*/d*h*. This causes a slow subsidence of the bed as the sediment is eroded. Since *σ* and *β* are small, this subsidence is essentially

4.4 $$b\approx -{\left(Au\right)}_{x}t,$$

corresponding to an erosion rate of the order of a millimetre per year. Note that then *s*_t_≈−*δ*(*Au*)_x_.

This basic state is unstable, analogously to the result of Fowler [6], and this can be due to the term −*σ*(*BSh*)_x_ in (4.1c). In [6], the basic state was taken as uniform (*h*=*const*.) and the slope *S* was also constant, but allowance was made for the nonlinearity of *B*: instability occurs if *B*′>0. This is analogous to the Smith-Bretherton situation [45], but we can directly see that instability will still occur if *B* is constant, if also the slope increases down flow (*S*′>0), which it sometimes, but not always, does in practice [46,47]. Instability is also promoted by the till flux term *Au***i**, if *u* increases with *x* as is likely, and *A* increases with *h*, i.e. *A*′(*N*)<0 (note this condition suppresses the ribbed moraine instability [42]).

Our specific concern is with the evolution of this instability as *h* grows to larger depths. When this occurs, it is clear from (4.1) that it is necessary to rescale *y*, and specifically, we write

4.5 $$y=\sqrt{\beta }Y,$$

corresponding to a length scale of about 1500 m, somewhat larger than the MSGL width scale found by Fowler [6]. We substitute this into (4.1), and then neglect the small terms in *ε*. We use the fact that *Ω*≫1 to neglect the advective term in (4.1e). We also neglect the small bedload transport terms in *σ*. This is inadmissible if true separation of the water film occurs, since then *A*=*N*=0, so we initially restrict our attention to the case where this is not so. We then obtain the approximating system

4.6a $$\begin{array}{r} \Psi =s-N+\Phi , \end{array}$$

4.6b $$\begin{array}{r} {\left[h^{3}\Psi_{Y}\right]}_{Y}=\frac{\beta }{\nu }\{{\left(Sh^{3}\right)}_{x}-1\}, \end{array}$$

4.6c $$\begin{array}{r} h_{t}=W+{\left(uA\right)}_{x}+{\left(A^{3}|N^{\prime }|h_{Y}\right)}_{Y} \end{array}$$

4.6d $$\begin{array}{r} \text{and}\;s_{t}=\delta W=\frac{\gamma \alpha }{\pi }\int_{-\infty }^{\infty }K_{0}(\alpha |Y-\eta |)\Phi (x,\eta ,t)\;d\eta , \end{array}$$

where

4.7 $$\alpha =a\sqrt{\beta },\;\gamma =\frac{2\Lambda }{a\Omega },$$

and

4.8 $$N=\frac{N^{*}(h)}{\Pi },\;A=\frac{1}{2}{\left[\frac{SN^{b}}{\mu {\bar{N}}^{b}}-N\right]}_{+}.$$

The values of the parameters *α* and *γ* are roughly *O*(1), as indicated in table 3. The reduction of the convolution integral in (4.1e) to the form in (4.6d) uses the fact that *Φ* varies relatively slowly in *x* compared with *y*, and then that the integral

4.9 $$\int_{|y|}^{\infty }\frac{e^{-ar}\;dr}{\sqrt{r^{2}-y^{2}}}=K_{0}(a|y|),$$

where *K*_0_ is the modified Bessel function of the second kind.

To be specific, we aim to solve the model (4.6) in the domain −*L*<*Y* <*L*, with boundary conditions of no sediment or water flow through the sides:

4.10 $$h_{Y}=\Psi_{Y}=0\;\mathrm{at}\;Y=\pm L.$$

It is a consequence of this that although *β*/*ν* is small in (4.6b), integration across the catchment yields the conservation law for water flow,

4.11 $$\frac{1}{2L}\int_{-L}^{L}Sh^{3}\;dY=x,$$

and we need to solve this in conjunction with (4.6c).

### (a) A reduced model

In this model, dependence on *x* is introduced only through the water flux (4.11), the advective till flux term in (4.6c) and the dependence of *A* on the slope in (4.8). The role of the *x* derivatives is to provide for change in mean water film depth (via (4.11)) and to provide an advection term for *A* (and thus *N* and thus *h*); neither of these has any essential effect on the mature downslope solution, and we therefore now focus on solutions which depend only on *Y* and *t*. It is possible to choose forms for *S* and *u* such that this is quantitatively justified (e.g. increasing downstream slope *S*=*x* and linearly increasing velocity *u*=*u*_0_+*x*), but we emphasize that such choices are only cosmetic. Actual surface slopes of ice streams are quite variable [46,47], but are generally more concave (thus decreasing slope) than the typical inland ice convex profile.

We can then take the approximate solution of (4.6b) to be *Ψ*≈0, in view of our earlier remark concerning the definition of *ψ* up to addition of an arbitrary function of time, so that the normal stress term

4.12 Φ≈N−s,

and the model simplifies with *u*_x_=1 to

4.13a $$\begin{array}{r} h_{t}=W+A+{\left(A^{3}|N^{\prime }|h_{Y}\right)}_{Y} \end{array}$$

4.13b $$\begin{array}{r} s_{t}=\delta W=\frac{\gamma \alpha }{\pi }\int_{-\infty }^{\infty }K_{0}(\alpha |Y-\eta |)[N\{h(\eta ,t)\}-s(\eta ,t)]\;d\eta , \end{array}$$

4.13c $$\begin{array}{rl} \text{and}\; & \frac{1}{2L}\int_{-L}^{L}h^{3}\;dY=1. \end{array}$$

A simpler version of the evolution equation for *s* results from a formal assumption of large *α*; then the spiky nature of *K*_0_(*α*|*Y* |), together with the fact that

4.14 $$\int_{-\infty }^{\infty }K_{0}(\alpha |Y|)\;dY=\frac{\pi }{\alpha },$$

suggests that we take *K*_0_(*α*|*Y* |)≈(*π*/*α*)*δ*(*Y*), and this leads to

4.15 $$s_{t}=\delta W=\gamma (N-s),$$

as a further simplification of (4.13b), and we shall use this as a basis for discussion. The implication of (4.15) or (4.13b) is that the basal ice surface and hence the bed relaxes to a state determined by the effective stress, itself determined by the water film depth; and where the film is thinnest (so *N* is high) the bed is most elevated.

The simplest discussion of the model (4.13), but using (4.15), assumes that *s* has relaxed to its equilibrium *s*=*N*(*h*), so that *W*=0 and (4.13) reduces to the system

4.16 $$\begin{array}{rl} & h_{t}=A+{\left(A^{3}|N^{\prime }|h_{Y}\right)}_{Y},\\ \text{and}\; & \frac{1}{2L}\int_{-L}^{L}h^{3}\;dY=1. \end{array}\}$$

The precise choices for the functions *N*(*h*) and *A*(*N*) are immaterial for the finite amplitude solutions that we obtain, and for ease of exposition we will take *A*=|*N*′|=1, not the least because it is by no means clear that (4.16) even has a solution. The partial differential equation for *h* paradoxically describes sediment conservation, and the two terms on the right represent the downstream sediment flux dragged by an accelerating ice flow, and the squeezing of subglacial till due to lateral variations in the effective stress, respectively; the integral term represents conservation of the quantity of water in the film.

We thus consider the following reduced model:

4.17 $$h_{t}=1+h_{YY},$$

together with

4.18 $$\begin{array}{rl} & h_{Y}=0\;\mathrm{on}\;Y=\pm L\\ \text{and}\; & \frac{1}{2L}\int_{-L}^{L}h^{3}\;dY=1. \end{array}\}$$

Because physically *h*≥0, the differential equation (4.17) should be interpreted as applying when *h*>0.

A different limitation occurs if *N* reaches zero, which will be the case if *h* reaches a critical value *h*_c_ where proper flow separation occurs: the ice is afloat and the water film becomes a stream or cavity. In this case, *A*=*W*=0 also and the model breaks down. It is in this situation where the bedload terms in *σ* may become important. We initially ignore this possibility, formally supposing that *N*>0 everywhere.

It is obvious that the model (4.17) and (4.18) has a fundamental problem, and this is not caused by any of our simplifications, which are inessential. The solution of the differential equation with the boundary conditions is inconsistent with the integral constraint. The problem lies with the latent assumption that *h*>0. As discussed above, the model for *h* is more properly

4.19a $$\begin{array}{r} h_{t}=1+h_{YY}\;\text{or}\;h=0, \end{array}$$

4.19b $$\begin{array}{r} h_{Y}\to 0\;\mathrm{on}\;Y=\pm L\;\mathrm{or}\;h=0\;\mathrm{on}\;Y=\pm L \end{array}$$

4.19c $$\begin{array}{rl} \text{and}\; & \frac{1}{2L}\int_{-L}^{L}h^{3}\;dY=1. \end{array}$$

It is clear that the solution must immediately contain regions of finite support, and that there is a steady state consisting of a number of streams. The simplest solution consists of a single stream, for which the steady symmetric solution is

4.20 $$\begin{array}{rl} h & =\frac{1}{2}(Y_{m}^{2}-Y^{2}),\;|Y|<Y_{m},\\ \text{and}\;h & =0,|Y|>Y_{m}, \end{array}\}$$

where

4.21 $$Y_{m}={\left(\frac{35L}{2}\right)}^{1/7},$$

and we must have

4.22 $$L>{\left(\frac{35}{2}\right)}^{1/6}\approx 1.61,$$

so that *L*>*Y*_m_. In the present situation, the meaning of the inequality is unclear, but its interpretation emerges in consideration of more general versions of (4.13a), where for example the choice *W*+*A*=*h*−1 shows that the inequality necessary for channel formation corresponding to (4.22) is precisely the condition for instability of the uniform steady state.

### (b) Numerical solutions

The only parameter in (4.19) is *L*, but it can be scaled out in the following way. Because the solutions develop finite support, the upper limit in the integral is irrelevant, and by rescaling

4.23 $$Y,t∼Y^{*},\;h∼Y^{*2},$$

the equation for *h* is unaffected, and the integral (assuming a symmetric solution) takes the form

4.24 $$\int_{0}^{L/Y^{*}}h^{3}\;dY=\frac{L}{Y^{*7}}.$$

A canonical form is chosen by selecting

4.25 $$Y^{*7}=L,$$

and in the canonical form of the problem, the integral constraint is

4.26 $$\int_{0}^{Y_{f}}h^{3}\;dY=1,$$

where *Y*_f_ is the stream boundary. The steady-state value of *Y*_f_ corresponding to (4.21) is

4.27 $$Y_{f}={\left(\frac{35}{2}\right)}^{1/7}\approx 1.505.$$

We now solve the canonical problem numerically. To do this, we follow a method devised by Fowler *et al*. [48] , where a very similar problem arises. First, we suppose the solution of (4.19) is symmetric, so that

4.28 $$h_{Y}=0\;\mathrm{at}\;Y=0.$$

Next, we change variables from *Y*,*t* to *X*,*T* defined by

4.29 $$T=t,\;X=\int_{0}^{Y}h^{3}(y,t)\;dy.$$

The chain rule gives

4.30 $$\frac{\partial }{\partial t}=\frac{\partial }{\partial T}+X_{t}\frac{\partial }{\partial X},\;\frac{\partial }{\partial Y}=h^{3}\frac{\partial }{\partial X},$$

and thus also, applying these to the inverse function *Y* (*X*,*T*),

4.31 $$1=h^{3}Y_{X},\;0=Y_{T}+X_{t}Y_{X}.$$

Substituting these into (4.19), and writing *T*=*t*, we obtain the pair

4.32a $$Y_{X}=\frac{1}{h^{3}},$$

and

4.32b $$h_{t}-h^{3}Y_{t}h_{X}=1+h^{3}{\left(h^{3}h_{X}\right)}_{X}.$$

We define

4.33 $$Y_{t}=w,\;X_{f}=1,\;{\left(X_{f}-X\right)}^{1/4}=\xi_{f}-\xi ,\;\xi_{f}=X_{f}^{1/4};$$

the variable *ξ* is a strained coordinate which is useful in improving the analyticity of the solution. We then obtain an equation for *w* by differentiating (4.32a) and substituting for *h*_t_ from (4.32b). This gives

4.34a $$\begin{array}{rl} h_{t} & =\phi , \end{array}$$

4.34b $$\begin{array}{rl} \phi & =1+\frac{w}{4}{\left(\frac{h}{\xi_{f}-\xi }\right)}^{3}h_{\xi }+\frac{1}{16}\left[{\left(\frac{h}{\xi_{f}-\xi }\right)}^{3}\frac{\partial }{\partial \xi }\left\{{\left(\frac{h}{\xi_{f}-\xi }\right)}^{3}\frac{\partial h}{\partial \xi }\right\}\right] \end{array}$$

4.34c $$\begin{array}{rl} \text{and}\;w_{\xi } & =-\frac{12{\left(\xi_{f}-\xi \right)}^{3}\phi }{h^{4}}, \end{array}$$

and the boundary conditions are

4.35 $$\begin{array}{rl} w & =h_{\xi }=0\;\mathrm{at}\;\xi =0\\ \text{and}\;h & =0\mathrm{at}\;\xi =\xi_{f}, \end{array}\}$$

the second of which implies *ϕ*=0 at *ξ*=*ξ*_f_. Furthermore, this front position *Y*_m_, where *h*=0, is defined by

4.36 $${\dot{Y}}_{m}=w(\xi_{f},t).$$

To solve (4.34), we step forward *h* explicitly, and then calculate *w* by quadratureQ2. Although the equations appear degenerate, a local analysis of the behaviour near *ξ*=*ξ*_f_ shows that

4.37 $$\begin{array}{rl} h & =\alpha (t)(\xi_{f}-\xi )+\frac{4(w_{f}\alpha^{4}-4)}{5\alpha^{6}}{\left(\xi_{f}-\xi \right)}^{2}+\cdots ,\\ \text{and}\;w & =w_{f}+\frac{12\dot{\alpha }}{\alpha^{4}}(\xi_{f}-\xi )+\cdots , \end{array}\}$$

where *w*_f_=*w*(*ξ*_f_,*t*).

There is some awkwardness in making the (finite difference) approximation near *ξ*=*ξ*_f_. Define

4.38 $$A={\left(\frac{h}{\xi_{f}-\xi }\right)}^{3},\;J={\left(\frac{h}{\xi_{f}-\xi }\right)}^{3}\frac{\partial h}{\partial \xi },\;\xi <\xi_{f}$$

and

4.39 $$A=-h^{\prime 3},\;J=-h^{\prime 4},\;h^{\prime }=h_{\xi }(\xi_{f}).$$

The definition of *ϕ* in (4.34) is thus

4.40 $$\phi =1+\frac{1}{4}wAh_{\xi }+\frac{1}{16}AJ_{\xi },\;J=Ah_{\xi },$$

and is approximated in a simple finite difference scheme by

4.41 $$\begin{array}{rl} \phi_{i} & =1+\frac{1}{4}w_{i}A_{i}\left(\frac{h_{i}-h_{i-1}}{\Delta }\right)+\frac{1}{16}A_{i}\left(\frac{J_{i}-J_{i-1}}{\Delta }\right),\\ \text{and}\;J_{i} & =A_{i}\left(\frac{h_{i+1}-h_{i}}{\Delta }\right),\;i=1,\ldots ,K-1, \end{array}\}$$

where Δ is the step size and *ξ*_f_=*K*Δ. The backward difference for the advective term is used if *w*_i_>0, otherwise the forward difference is used. In an explicit method, we only need to evaluate *ϕ* for steps 1 to *K*−1, but the quadrature for *w* also needs *ϕ* at *i*=0. From (4.40), we have $\phi =\frac{1}{16}A^{2}h_{\xi \xi }$, and therefore, using a 3-point approximation for *h*_*ξξ*_ at *ξ*=0 (bearing in mind that *h*_*ξ*_=0 there, so *h*_0_≈*h*_1_), we take

4.42 $$\phi_{0}=1+\frac{A_{0}^{2}(h_{2}-h_{1})}{24\Delta^{2}}\;\mathrm{and}\;A_{0}={\left(\frac{h_{1}}{\xi_{f}}\right)}^{3}.$$

The prescription of *ϕ*_*K*−1_ from (4.41) is immediate, even if *w*_*K*−1_<0, since we have *h*_K_=0. An alternative strategy uses the fact that *ϕ*=0 at *ξ*=*ξ*_f_, together with (4.39), to show that

4.43 $$J^{\prime }={\frac{\partial J}{\partial \xi }|}_{\xi =\xi_{f}}=-4w_{f}h^{\prime }\;\mathrm{at}\;\xi =\xi_{f};$$

we could then replace (4.41) by

4.44 $$\begin{array}{rl} \phi_{i} & =1+\frac{1}{4}w_{i}A_{i}\left(\frac{h_{i}-h_{i-1}}{\Delta }\right)+\frac{1}{16}A_{i}\left(\frac{J_{i+1}-J_{i}}{\Delta }\right)\\ \text{and}\;J_{i} & =A_{i}\left(\frac{h_{i}-h_{i-1}}{\Delta }\right),\;i=1,\ldots ,K-1, \end{array}\}$$

except that for *i*=*K*−1, we replace the approximation of *J*′ by

4.45 $$J^{\prime }=\frac{4w_{f}h_{K-1}}{\Delta }.$$

**Figure 2.**
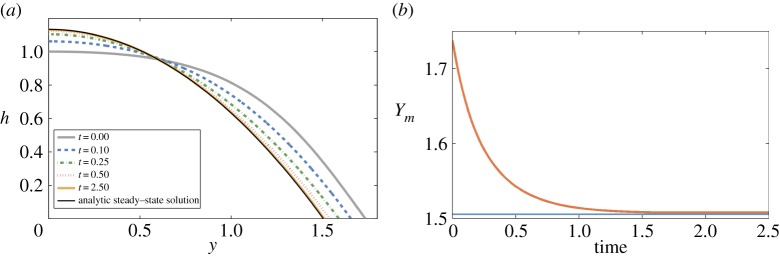
Solution of (4.19), but using (4.26), small perturbation from steady state. (Online version in colour.)

**Figure 3.**
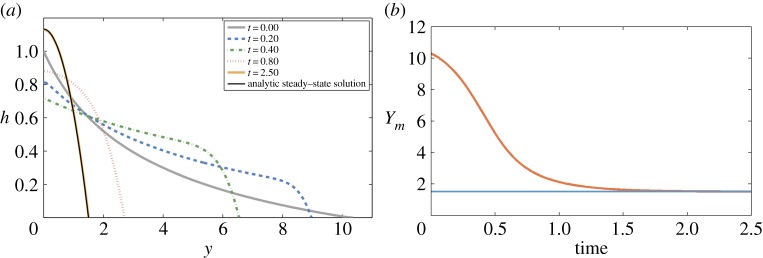
Solution of (4.19), but using (4.26), large perturbation from steady state. (Online version in colour.)

Figures 2 and 3 give two typical numerical solutions of (4.19) (in the canonical version with (4.26)). The solution in figure 2 is initialized with a small perturbation from steady state and that for figure 3 a much larger perturbation. It can be seen that both relax to the steady state as time progresses.

## 5. Discussion

The derivation of (4.19) from the reduced model (4.13) assumed that *W*=0, based on the equilibrium for *s* in (4.15). If instead we start from an ice flow which is out of equilibrium, for example, a uniform state with *s*=0, then (4.15) suggests

5.1 $$W=\frac{w}{\delta },\;w∼O(1),$$

and a rescaling of (4.13a) is then necessary. Taking the diffusion coefficient to be one, this is (bearing in mind the integral constraint and presuming that *L*∼*O*(1))

5.2 $$h∼\frac{1}{\delta^{1/7}}∼3.43,\;Y∼\delta^{3/7}∼0.025,\;t∼\delta^{6/7}∼0.62\times 10^{-3},$$

corresponding to new depth, breadth and time scales describing the size and evolution of streams of 1.3 cm, 38.5 m and 2.3 days. With this rescaling, the model takes the form (using (4.15)):

5.3 $$\begin{array}{rl} & h_{t}=w+\delta A+h_{YY},\\ & s_{t}=\delta^{6/7}w,\;w=\gamma (N-s),\\ \text{and}\; & \int_{-\infty }^{\infty }h^{3}\;dY=2L. \end{array}\}$$

The results of solving this are essential as before, except that the streams are a little deeper and less wide: on the short 2-day time scale, *s*≈0 is stationary and *h* tends to stationary finite width stream solutions of *h*_*YY*_+*γN*(*h*)=0.

In the final approach to the steady state, we have *s*≈*N*(*h*), and thus more accurately

5.4 $$W=\frac{s_{t}}{\delta }=-\frac{|N^{\prime }|h_{t}}{\delta }.$$

In that case, the model takes the form, with the original scales,

5.5 $$\left(1+\frac{|N^{\prime }|}{\delta }\right)h_{t}=A+{\left(A^{3}|N^{\prime }|h_{Y}\right)}_{Y},$$

together with the integral constraint, and the result is as before, albeit with the evolution on the much longer time scale of ice flow adjustment: the evolution of the ice roof acts as a huge brake on the development of the fluvial system.

Two complications merit discussion in our simple analysis of (5.5), or (4.13a), where we took *A* and |*N*′| as constant, and these are the consequences of taking more realistic forms for these functions.

The first concerns what happens as *h*→0. In reality, water is produced everywhere at the bed from the ice above and so the film thickness can never truly go to zero. This is manifested in the model through the closure relation (see (2.24)), which implies that *N*(*h*)∝(l*)^−1^, where l* is the supporting clast spacing. Effectively, this tends to zero as *h*→0, implying that N→∞, and this prevents *h* reaching zero. In addition, the deformable till thickness *A* in (3.6) becomes zero for *N* larger than a critical *O*(1) value, and so the bed becomes immobile, *b* is constant, and the water film thickness is determined geometrically from the solution of *s*=*N*(*h*)=*h*, assuming *b*=0.

Of apparently greater interest is what happens if *N* reaches zero, when also *A*=0. When this occurs, the ice surface separates properly from the bed and a stream or cavity forms. We consider briefly the dynamics of such streams. If *N*=0 the till flux and squeezing terms vanish, and the fluvial sediment transport terms become significant, on a longer time scale. If we bring back the fluvial terms in (4.1c) and ignore the till flux and squeeze terms, we find (again taking *s*=*N* and thus =0), that (5.5) is modified to

5.6 $$\frac{1}{\sigma }h_{t}=h-{\left[\frac{1}{h^{2}}\int_{0}^{Y}(h^{3}-1)\;dY\right]}_{Y}+\frac{\lambda }{\beta }h_{YY},$$

where the integral term arises from the solution of (4.6b) correct to *O*(*β*/*ν*), and we have assumed a symmetric solution in which there is no water flow across the divide at *Y* =0; we have also taken for illustration (*BS*)′=1, although other choices are possible (in particular, we could have (*BS*)′<0 for a concave upper ice surface) [46,47]. The three terms on the right represent excavation of the bed by increasing downflow bedload transport, cross-stream bedload transport, and downbedslope sediment rolling, respectively.

Note that λ/*β*∼10^−2^, so that the natural length scale for the stabilizing bedslope term in (5.6) is

5.7 $$Y=\sqrt{\frac{\lambda }{\beta }}Z,$$

corresponding now to a width of 166 m. We assume that *h* reaches a steady state, whose profile thus represents stream depth, and these steady solutions of (5.6) satisfy

5.8 $$0=h-{\left[\frac{1}{h^{2}}\int_{0}^{Z}(h^{3}-1)\;dZ\right]}_{Z}+h_{ZZ}.$$

**Figure 4.**
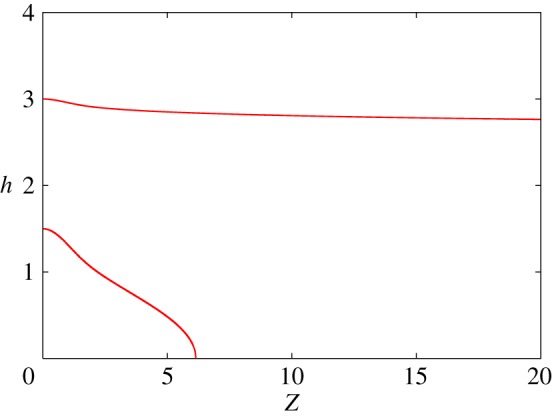
Solutions of (5.8) with *h*_Z_=0, *h*=*h*_0_ at *Z*=0, for *h*_0_=1.5,3. (Online version in colour.)

Figure 4 shows numerical solutions of this equation, assuming a symmetric solution, and for various values of the centre-line dimensionless depth *h*_0_. A comment on the method of solution is necessary. We solve the equation by writing it in the form of a system

5.9a $$\begin{array}{rl} q^{\prime } & =h^{3}-1, \end{array}$$

5.9b $$\begin{array}{rl} h^{\prime } & =\frac{q}{h^{2}}-w \end{array}$$

5.9c $$\begin{array}{rl} \text{and}\;w^{\prime } & =h, \end{array}$$

with boundary conditions *q*(0)=*w*(0)=0, *h*(0)=*h*_0_.

Of particular interest is whether significantly deep streams can be predicted from this theory: these would correspond to large numerical values of *h*_0_≫*h*_c_, where *h*_c_ is the presumed critical value, where *N* reaches zero and separation occurs. Figure 4 indicates that as the maximum stream depth increases, the solution of (5.8) becomes approximately constant,^1^ which suggests that large depth streams of constant depth are only possible providing *h*_c_≫1. However, this appears to require the presence of coarse clasts, which seems unlikely under the Ross ice streams. On the other hand, the prescription of a local clast spacing law as in (2.24) does not seem likely when streams form, and further discussion of the formation of genuine metre deep streams may require further elaboration of the film closure model.

## 6. Conclusion

In this paper, we have endeavoured to provide a mechanism for subglacial stream formation based on a first principles description of the interaction of ice, water and sediment. The results of our study suggest that for a Creyts–Schoof water film of thickness approximately 5 mm beneath an ice sheet, a uniform film is unstable, just as is the case for subaerial overland flow, and the model suggests that ‘streams’ of finite width will develop. While details will depend on the precise physical conditions, these streams appear to be more like swamps, having widths of hundreds of metres and depths of the order of a centimetre. Such a style of drainage is consistent with Alley's [10] notion of a patchy film beneath ice streams, and it is also consistent with Engelhardt & Kamb's [49] direct borehole observations beneath the Kamb Ice Stream of an ice/till gap of 1–2 cm in their borehole 5.

What we are less able to comment on is the observation by Engelhardt and Kamb of a 1.4-m deep water layer at borehole 9, indicative in their view of a subglacial channel. An alternative possibility is that this layer may represent a subglacial cavity formed by a developing drumlin field following the shutdown of the ice stream, but the issue remains as to the origin of the water. Concentration of an average 5 mm thick water film into a 1.4-m deep channel represents an amplification of 280 and such a stream of width, e.g. 5 m would drain all the water from subglacial melt from a surrounding width of 1400 m. While this seems feasible, our theory does not apparently provide the mechanism for such deep streams. On the other hand, the water-saturated till itself contains a large quantity of water: 8 m of till with porosity 0.4 has an effective film depth of 3.2 m! Following the Kamb Ice Stream's shutdown, the basal effective pressure would have risen, and this allows the water to be sucked out of the till. In that case, it would be possible to explain the presence of pockets of water, though not within the present theory, which ignores groundwater storage (cf. [50]). It is actually not difficult to include till storage in our model, allowing for infiltration to, or expulsion from the water film as the effective stress varies locally. However, the effect of the till is to act as a giant sponge, with the only dynamic effect being to provide a longer time scale for film thickness adjustment; essentially, this is because there is no net source associated with the till layer, it simply acts as a reservoir.

Even though the model is complicated, it does introduce simplifications that can be criticized. Examples of these are the assumptions of a constant viscosity for ice, a rheology for till deformation that assumes a yield stress based on solid friction and a prescribed effective viscosity, and the use of a simple linear bedload sediment transport law; however, in our view the most critical component of the model is the prescription of a closure relation between effective pressure and water film thickness, and it seems probably that more refined descriptions of this will be made in the future.

So what perspective does the present theory provide for present-day subglacial drainage? One of the things we know about subglacial water, at least beneath the Antarctic, is that there are numerous lakes, and that these form an ingredient of an active drainage system, in which lakes sporadically drain from one to the other [1,2,51]. It seems possible that basal water flows slowly through a patchwork of swamps, with occasional floods opening larger channels, although the dynamics of such floods remains to be elucidated. A further possibility, and one suggested by recent work of Christoffersen *et al.* [50], is that groundwater stored in subglacial sediment acts as a storage reservoir, which in conditions of stream formation allows expulsion of groundwater at topographic highs (where *s*≈*N* is large, *h* is small), but further examination of this possibility requires a specific inclusion of groundwater storage into the model.

The overriding motivation for studying the subglacial water system lies in a bid to explain feedbacks with the ice sheet flow. In this paper, we have concentrated on modelling on a small scale, with feedbacks from the water to the ice flow not included. Recent work by the authors has considered the feedbacks for a simplified version of the system presented here (not including sediment evolution) [41], but there is scope for a lot of work to be done in the future considering these feedbacks in more detail. Upscaling a model such as that presented here can be further used as a basis to develop subglacial water flow parametrizations for large-scale ice-sheet models.

## Footnotes

1 This is consistent with an asymptotic approximation for large *h*_0_, which follows straightforwardly by rescaling *h*∼*w*∼*h*_0_, $q∼h_{0}^{3}$ and expanding in powers of $\varepsilon =1/h_{0}^{3}$, from which one can find the regular expansion for the rescaled h∼1−121εZ+14επ erf Z, or with a multiple scale approach, $h∼{\left(1-\frac{3}{2}\varepsilon Z\right)}^{1/3}+(\varepsilon /2{\left(1-\frac{3}{2}\varepsilon Z\right)}^{2/3})\int_{0}^{Z}F(y)\;dy$, where $F(y)=e^{-y^{2}}\int_{0}^{y}\;e^{t^{2}}\;dt$ is Dawson's integral. Evidently, this latter expansion must itself be adjusted using strained coordinates where *h*→0.
